# A Randomized Controlled Pilot Study of Home-Based Step Training in Older People Using Videogame Technology

**DOI:** 10.1371/journal.pone.0057734

**Published:** 2013-03-05

**Authors:** Daniel Schoene, Stephen R. Lord, Kim Delbaere, Connie Severino, Thomas A. Davies, Stuart T. Smith

**Affiliations:** 1 Falls and Balance Research Group, Neuroscience Research Australia, Sydney, New South Wales, Australia; 2 School of Public Health and Community Medicine, University of New South Wales, Sydney, New South Wales, Australia; Charité University Medicine Berlin, Germany

## Abstract

**Background:**

Stepping impairments are associated with physical and cognitive decline in older adults and increased fall risk. Exercise interventions can reduce fall risk, but adherence is often low. A new exergame involving step training may provide an enjoyable exercise alternative for preventing falls in older people.

**Purpose:**

To assess the feasibility and safety of unsupervised, home-based step pad training and determine the effectiveness of this intervention on stepping performance and associated fall risk in older people.

**Design:**

Single-blinded two-arm randomized controlled trial comparing step pad training with control (no-intervention).

**Setting/Participants:**

Thirty-seven older adults residing in independent-living units of a retirement village in Sydney, Australia.

**Intervention:**

Intervention group (IG) participants were provided with a computerized step pad system connected to their TVs and played a step game as often as they liked (with a recommended dose of 2–3 sessions per week for 15–20 minutes each) for eight weeks. In addition, IG participants were asked to complete a choice stepping reaction time (CSRT) task once each week.

**Main Outcome Measures:**

CSRT, the Physiological Profile Assessment (PPA), neuropsychological and functional mobility measures were assessed at baseline and eight week follow-up.

**Results:**

Thirty-two participants completed the study (86.5%). IG participants played a median 2.75 sessions/week and no adverse events were reported. Compared to the control group, the IG significantly improved their CSRT (F_31,1_ = 18.203, p<.001), PPA composite scores (F_31,1_ = 12.706, p = 0.001), as well as the postural sway (F_31,1_ = 4.226, p = 0.049) and contrast sensitivity (F_31,1_ = 4.415, p = 0.044) PPA sub-component scores. In addition, the IG improved significantly in their dual-task ability as assessed by a timed up and go test/verbal fluency task (F_31,1_ = 4.226, p = 0.049).

**Conclusions:**

Step pad training can be safely undertaken at home to improve physical and cognitive parameters of fall risk in older people without major cognitive and physical impairments.

**Trial Registration:**

Australian New Zealand Clinical Trials Registry ACTRN12611001081909.

## Introduction

Falls are very common in older people [1]. Declines in physical and cognitive functioning, that have also been identified as intrinsic fall risk factors [2], [3], may lead to reduced capabilities for taking proactive and reactive steps in order to maintain balance [4], [5]. Impaired stepping has been associated with falls in older people in several studies [6], [7], [8]. In response to an external perturbation of balance, older fallers are more likely to: take an inappropriate step (wrong direction, too short), to collide one leg against the other during compensatory crossover steps [7], to be slower in initiating volitional step responses [6], and be distracted when stepping under dual task conditions [8].

Physical exercises that include a balance component can reduce falls in older people [9]. In order to be effective, it has been suggested that balance exercise should be undertaken with reduced base of support, minimized upper limb support and include weight shifting components such as stepping [9]. Some stepping exercises have been incorporated into existing exercise programs aimed at preventing falls, but only limited evidence is available on the isolated effect of stepping intervention towards falls prevention in older people. A few studies have demonstrated improvements in parameters of fall risk in older people, such as enhancements of balance, reaction time and reactive responses to unpredictable balance perturbations [10], [11], [12]. In addition, physical exercises may improve specific cognitive functions [13], [14], [15] that are associated with fall risk of older people.

Despite clear evidence demonstrating benefits of exercise for reducing fall risk, uptake and adherence to exercise programs in fall prevention is often disappointing [16], [17]. Efforts to improve exercise adherence are needed to increase the impact of falls prevention programs at a population level. One method by which compliance with exercise programs could be improved involves the use of enjoyable videogames. Interactive, exercise-based videogames (exergames) that combine player movement, engaging recreation, performance feedback and social connectivity via competition have been shown to promote motivation for, and increase adherence to, physical exercise amongst children and young adults [18], [19]. It has also been shown that certain exergames correspond to moderate intensity exercise in older people [20], [21]. Providing exergame technology to older people for home-based training could increase compliance to effective programs, potentially benefiting more people.

The purpose of this study is to explore the use of an exergame that requires participants to take steps on a step pad following instructions displayed on a screen (e.g. Dance Dance Revolution-DDR; Konami). This game type requires repetitive stepping in all directions, different speeds and provides different game complexities, challenging balance, coordination, reaction time and attention [22]. To our knowledge no RCT in older people using step pad games alone has been reported. The aims of this pilot study was a) to establish the feasibility and safety in administering an unsupervised, home-based step pad training intervention and b) the effectiveness of this intervention in improving stepping ability as well as physical and neuropsychological factors associated with falls in older people.

## Methods

The supporting CONSORT checklist is available as supporting information; see Checklist S1.

### Participants and Recruitment

Eligible participants were residents of independent-living units (ILUs) of a retirement village in Sydney aged 65 years or older, able to walk without a walking aid for 20 m, able to step in place unassisted on a step pad and without disabilities in ADL/IADL functions. Exclusion criteria were major cognitive impairment (MMSE<24), diagnosis of degenerative disease (e.g. Parkinson Disease), other health problems affecting stepping ability (e.g. acute painful joint inflammation, mobility impairment after stroke), or any unstable health conditions. All participants gave written informed consent prior to data acquisition. The study protocol was approved by the University of New South Wales’ Human Research Ethics Committee.

To detect an improvement in step reaction time of 15% ( = 158 ms SD150) in a population with an underlying step reaction time of 1056 ms (SD = 162) [23] with a two-sided significance level of 5% and 80% power, a sample size of 15 participants per group was necessary. With an anticipated drop-out of 25%, 37 participants were recruited.

Block-randomization was applied to form two groups of similar size. Couples (two people living in the same household) were treated as one unit and randomized into their own blocks to ensure that equal numbers of couples were allocated to intervention and control groups. Block size was four with one block of two. Randomization was undertaken for all participants at one point in time to avoid selection bias using a computer-generated list of random numbers by a staff member not involved in the study with group allocation provided by Email. Personnel administering assessments were also blinded to group allocation.

### Intervention

The open-source DDR game Stepmania (www.stepmania.com) was modified for the purposes of the study. A computer unit and step pad were installed in each intervention participant’s home. The computer unit was connected to participants’ television sets to control presentation of the intervention stimuli. The step pad connected to the computer unit consisted of pressure sensitive panels which represented stepping direction (arrows, stars), program control (“A” and “B”) as well as two central stance panels (cartoon feet). The computer unit recorded timing of foot lift and landing at each panel.

**Figure 1:**
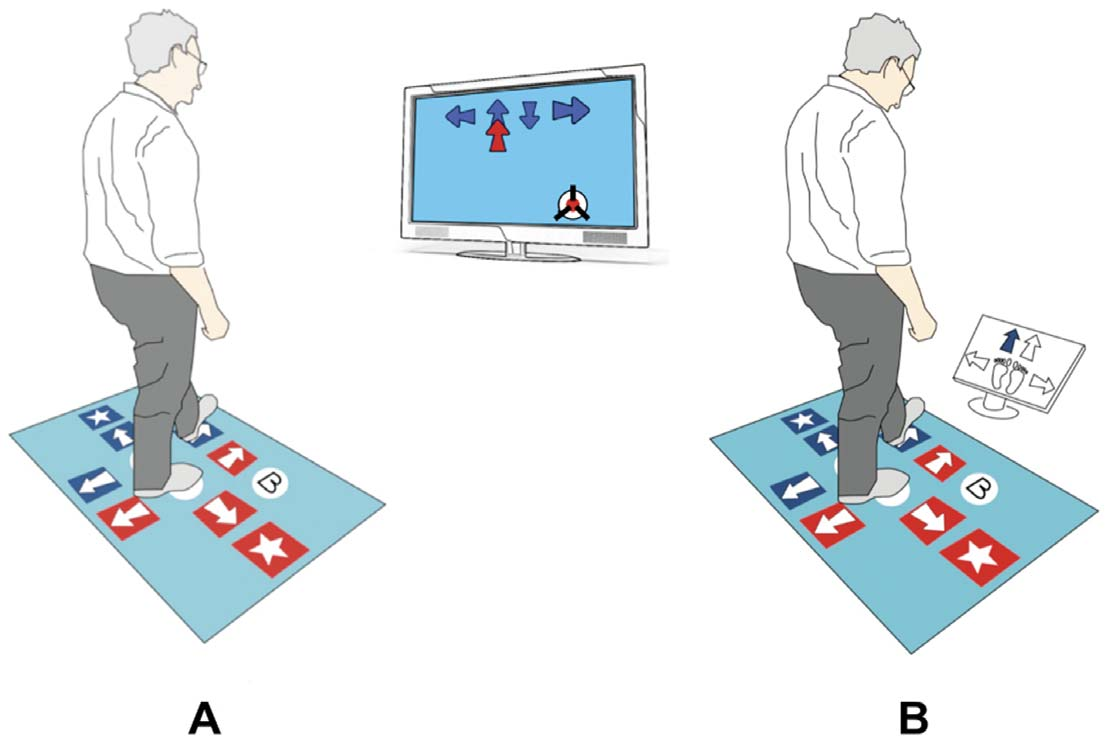
Step pad and intervention tasks used in this study. a) DDR example screen with updrifting arrow. The participant has to step when the red arrow (moving up) is directly over the target arrow (blue arrows). The round object on the bottom right side is a ‘bomb’ for which the participant has to inhibit his step. b) CSRT example screen. One of four arrows on the screen changes its color to blue and the participant is asked to step as quickly as possible onto the same location of the pad (front left).

The game required participants to step as accurately as possible, both in terms of direction and timing, while synchronizing their stepping with instructions presented on the screen. Arrows drifted from the bottom to the top of the screen (Figure 1 red arrow) and over a target arrow (Figure 1 blue arrows). Participants were asked to time each step so that it corresponded precisely with the drifting arrow passing over the target (Figure 1). After each step response, participants were required to return their stepping foot back to the appropriate central stance pad panel. For each step feedback was given in form of a word in the centre of the screen (perfect, good, miss). Points accrued according to how well participants performed the task.

To introduce an additional cognitive load to the intervention, at various points during the game, a ‘bomb’ (round object), was randomly presented instead of a drifting arrow. This required participants to inhibit their step response. If participants failed to avoid a bomb the object ‘exploded’ as an indication of the error and points were correspondingly deducted from their game score.

Participants played the game with accompanying music selected by participants. Stepping sequence patterns were not synchronized with the rhythm of the music. The game contained three levels of difficulty (easy, medium, hard), and each had a separate range of musical tracks. Levels differed in terms of speed of moving objects (between 3.2°/s to 6.3°/s for a standard television of 40 cm height and a viewing distance of 2 m), number of objects on the screen at the same time (two to four), and number of distracters (bombs, colors). It is important to note that the complexity of the DDR stimulus is defined not only by arrow drift rate (speed) but also the rate of stimulus presentation, i.e. how many steps/per second players are expected to make onto a target location in order to successfully interact with the game. Depending on the game difficulty, target step rate varied between one step every two seconds to one step each second. The overall number of steps and ‘bombs’ also increased (easy = 72 steps, 8 bombs; medium = 80 steps, 12 bombs; hard = 116 steps, 18 bombs). Participants were instructed to progress to a higher level when they considered they were performing well at their current level or considered the game level to be not sufficiently challenging and return to a lower level if they considered the game level was too difficult.

Participants were instructed how to use the system and play the stepping game in a 90 minute individualized session in their homes. In addition, they received a manual with step by step instructions and important advice regarding the control of the system and the training program. They were asked to play the game as many times as they wished with the recommended dose of 2–3 sessions per week for 15–20 minutes each session over the 8 weeks of the trial. The songs had durations of three to four minutes. The process of selecting and initiating songs provided participants with rest breaks within the exercise sessions. To enhance movement speed in addition to movement coordination they were asked to complete a choice stepping reaction time (CSRT) task once a week (described in outcome measures). Intervention group (IG) participants were contacted by phone in week 1, 2, 3 and 6 to facilitate compliance and assist participants with any difficulties they experienced using the system. Furthermore, participants were given a telephone number they could call if they required any further help.

Participants in the control group were asked to continue to perform usual activities during the eight weeks intervention period.

### Outcome Measures

Assessments, including a questionnaire on demographic and health status items (Table 1), were performed in a quiet room within the retirement village and all participants were assessed under the same conditions at baseline and within seven days following the completion of the eight week intervention.

**Table 1:** Baseline characteristics.

Variable	Intervention (n = 15)	Control (n = 17)	p-value
**Age (years)**	77.5±4.5	78.4±4.5	0.586
**MMSE**	28.9±1.1	28.8±1.1	0.912
**CSRT RT (ms)**	755±81	730±74	0.393
**CSRT MT (ms)**	252±44	245±44	0.672
**CSRT resp (ms)**	1007±115	975±104	0.447
**INHIB time 20trials**	50.8±17.2	53.1±10.6	0.650
**INHIB time/trial**	2.5±0.8	2.5±0.5	0.853
**INHIB errors**	1.0±1.9	1.1±1.5	0.844
**PPA**	1.75±0.64	1.55±0.82	0.438
**AST (s)**	9.2±1.8	9.3±4.7	0.683
**TUG (s)**	9.6±1.3	9.8±1.4	0.658
**TUG animal (s)**	14.1±5.6	11.9±2.9	0.161
**5STS (s)**	11.5±2.3	10.8±2.4	0.387
**TMT B-A (s)**	46.8±21.3	61.4±36.0	0.282
**Icon-FES**	16.3±4.5	17.6±5.6	0.534
**n fallen past year**	5	4	0.699
**n pain lower limbs**	10	10	0.647

AST = alternate step test; CSRT = choice stepping reaction time; Icon-FES = iconographical falls efficacy scale; MMSE = Mini-mental State Examination; MT = movement time; PPA = Physiological Profile Assessment score; resp = response time; RT = reaction time; TMT B-A = difference between Trailmaking test B and A; TUG = timed up & go test; 5STS = five times sit-to-stand.

The primary outcome measure was Choice Stepping Reaction Time (CSRT). Secondary outcome measures included physiological fall risk, physical and cognitive performance and falls efficacy.

#### Choice stepping reaction time

CSRT was measured in milliseconds using a step pad as described above. On the screen participants saw a graphical presentation of the arrows on the mat. The step direction was indicated by one arrow changing its color. Participants were asked to step as quickly as possible onto the corresponding arrow of the pad and returned to the centre (Figure 1b) [23]. The test consisted of 4 practice trials (one trial for each step direction: side left, front left, front right, side right) and 32 test trials with stimuli occurring randomly between 1 and 2 seconds after the participant returned to the centre. Participants were instructed not to anticipate stimuli location as stimulus presentation would be random. Time was measured in milliseconds and subdivided into: reaction time (RT) measured from stimulus occurrence to movement initiation (lift off); movement time (MT) measured from movement initiation to step finalization (step down); total response time measured as the sum of RT and MT [23].

#### Secondary outcome measures

Physiological fall risk was estimated using the Physiological Profile Assessment (PPA). This test battery includes five tests of different physiological functions involved in postural stability: contrast sensitivity, proprioception of the lower extremities (knee joint position sense), lower extremity strength (isometric knee extension), standing balance (postural sway on a compliant surface) and simple hand reaction time. A fall risk score is generated from these five weighted z-transformed sub-scores and a higher score indicates higher fall risk [24]. PPA fall risk is designated mild if the score is between 0 and 1, moderate between 1 and 2, and marked for scores >2 [24].

General physical performance was measured using the Timed up & go test (TUG), Sit to Stand 5 times (5STS) and Alternate Step test (AST). The TUG measures mobility by taking the time in seconds to get up from a chair, walk 3 meters, turn, return and sit down again [25]. 5STS is a measure of functional strength and was assessed by asking participants to stand up and sit down 5 times as fast as possible [26]. Functional balance was assessed using the AST during which participants placed their feet eight times in alternate order on a sturdy wooden step (18 cm high) as fast as possible [27].

Cognitive function was assessed using the Trail making test (TMT). TMT evaluates scanning, visuomotor tracking, divided attention, and cognitive flexibility. TMT consists of two parts, part A and part B. Time in seconds to complete the test was measured. The difference in execution time between TMT B and TMT A was computed because it is less dependent on visuomotor speed and gives a good estimate of executive function [28].

Dual task performance was assessed by asking participants to repeat the TUG while doing a simultaneous verbal fluency task (naming animals) requiring participants to name as many different types of animals as they could during the time it took them to complete the TUG. No instructions were given that would have led to prioritization of either task.

An additional stepping task (INHIB), incorporating an inhibitory component, was used to test the impact of executive function on dynamic balance. Participants stood in the centre of the step pad. In the centre of the screen an arrow was presented pointing in one of four directions (up, down, left, right). Inside the arrow was a written word in a high contrast color indicating a different direction. Participants were instructed to step according to the word and by doing that to selectively attend to one stimulus and inhibit the response indicated by the arrow’s shape. After 4 practice trials (one for each step direction: left, forward, right, backward), the total time taken to complete 20 trials, the mean trial time excluding erroneous trials, and the number of errors were measured. This test has been reported to discriminate between fallers and non-fallers [29].

Fear of falling was measured by the short version of the icon-FES, an iconographic questionnaire (10 items) showing pictures of daily activities that vary with respect to their balance demands. The participant was asked about perceived concerns on a four level Likert scale. Higher scores indicate higher levels of concern [30].

In addition, the safety of the step pad training intervention was measured through incidents reported by participants during phone interviews and by feedback questionnaires completed at the end of the intervention. This questionnaire also included a single question (yes/no) asking whether participants enjoyed playing the exergame.

Adherence was measured using recordings from the computers as well as from self-reports at the end of the intervention period.

### Statistical Analysis

For variables with skewed distributions, data were log-transformed and analyses were performed with the normalized data. Independent t-tests (for continuous data) and Chi square tests (for categorical data) were used to determine differences between the intervention and control groups at baseline. Analysis of covariance (ANCOVA) was used to compare the effect of the intervention on outcome measures at follow-up adjusting for baseline values. Possible dose-response relationships were explored using paired t-tests. The alpha level was set at 5%. Analyses were performed with SPSS (version 20 for Windows, IBM Corp.).

## Results

### Participant Recruitment and Retention

**Figure 2:**
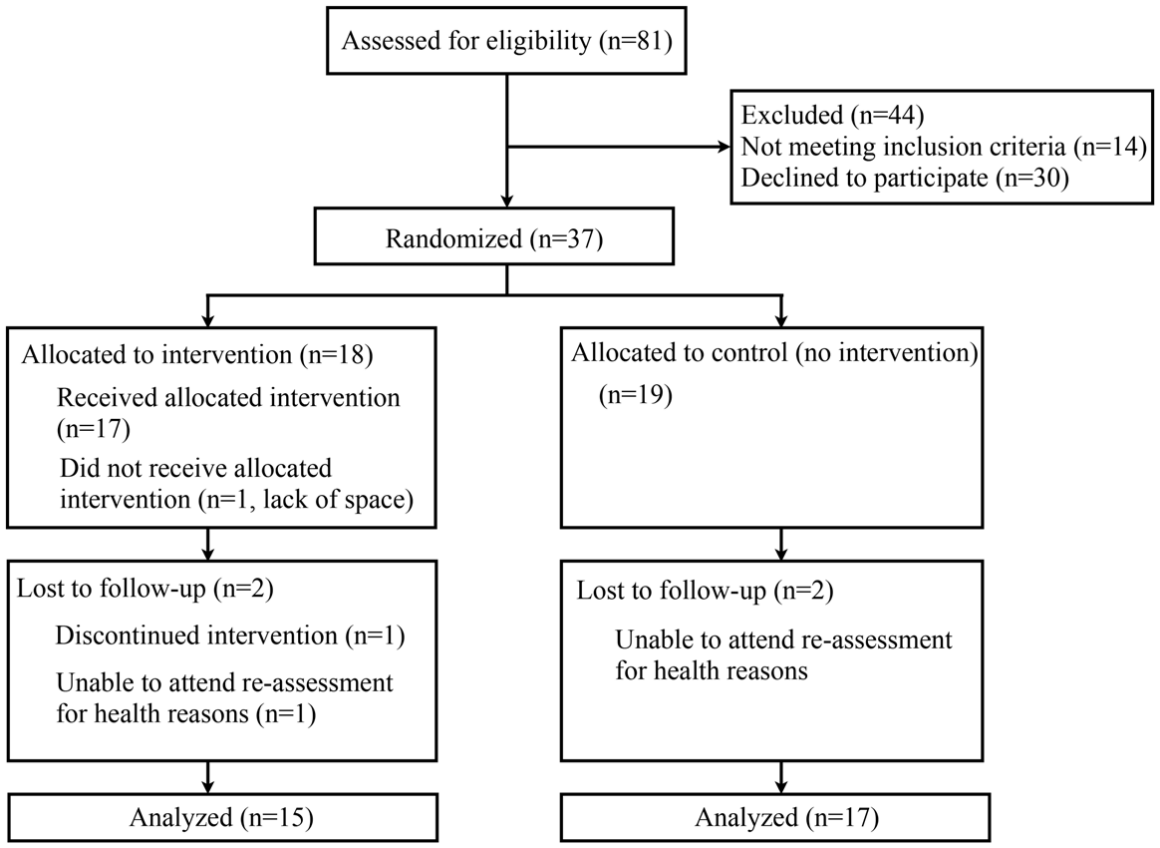
Flow chart of study.

Figure 2 shows the flow of participants through the study (August-December 2011). Eighty-one participants were screened. Of these, 30 declined participation in an intervention study and 14 were not eligible due to cognitive impairment (n = 1), diagnosis of a degenerative disease (n = 1) or other health problems affecting their ability to step safely without supervision. Thirty-seven people were randomized into the intervention (IG n = 18) and control groups (CG n = 19). Thirty-two participants (15 IG, 17 CG; 78±5 years, min-max 69–85years) completed the study, which corresponds to an overall drop-out rate of 13.5%. The withdrawal rate was similar for both groups. One person did not receive the intervention due to limited available space in the home. Another three participants (one IG participant, two CG participants) could not attend the re-assessment due to health reasons unrelated to the intervention and were excluded from the analysis. One participant was unable to use the system. Intervention and control groups were well-matched for baseline demographic, health, mobility and lifestyle characteristics, as presented in Table 1.

### Adherence, Progression, Adverse Events and Enjoyment

Fifteen intervention participants used the step training system throughout the eight weeks intervention period. The median number of sessions played per week was 2.75 (IQR = 2.25–3.15) with a median duration of 15 minutes (IQR = 11–22.5) per session. Participants completed the additional CSRT seven times (IQR = 5–8.5) during the intervention period. All but one participant increased the difficulty level of the game independently. At the end of week one, 12 participants (80%) were playing at the easy level, whereas at the end of the intervention 8 participants (53%) were playing at the hard level, 5 (33%) at the medium level and only 2 (13%) at the easy level (one of which had played levels medium and hard in between). All participants played more than one song. No adverse events related to the intervention were reported. All but one participant indicated they enjoyed the intervention.

### Effects of the Intervention

ANCOVAs revealed significant between-group differences at re-assessment in step reaction time (F_31,1_ = 14.44, p = 0.001), step movement time (F_31,1_ = 6.285, p = 0.018), overall step response time (F_31,1_ = 18.203, p<0.001) in favor of the IG. Overall 14 intervention participants improved step reaction and movement times. IG participants also demonstrated significantly greater improvement than control participants on the PPA composite score (F_31,1_ = 12.706, p = 0.001), as well as on the postural sway (F_31,1_ = 4.226, p = 0.049) and contrast sensitivity sub-component (F_31,1_ = 4.415, p = 0.044) scores. In addition, the IG improved significantly in the dual-task TUG-animal test (F_31,1_ = 4.226, p = 0.049) and showed trends for improved INHIB stepping time performance (F_31,1_ = 3.004, p = 0.094). Overall, 13 intervention participants improved their PPA score, sway sub-scores and on the dual-task TUG times. There were no between-group differences for any of the other outcome measures (Table 2).

**Table 2:** Results of DDR stepping on outcome measures.

Item	Groups	Baseline Mean (SD)	Re-assessment Mean (SD)	Group x time interaction (p-value)	% change from baseline^a^
**CSRT RT**	DDR	755±81	679±67	**.001**	10
	CON	730±74	738±92		−1
**CSRT MT**	DDR	252±44	210±47	**.018**	17
	CON	245±44	241±63		2
**CSRT resp**	DDR	1007±116	890±97	**.000**	12
	CON	975±104	979±134		0
**PPA**	DDR	1.75±0.6	1.15±0.8	**.001**	34
	CON	1.55±0.8	1.56±0.8		−1
**Sway path**	DDR	386±132	301±133	**.049**	22
	CON	355±118	330±95		7
**Sway AP**	DDR	44±16	34±13	.577	23
	CON	36±11	32±9		11
**Sway ML**	DDR	53±21	33±16	.139	38
	CON	38±15	36±16		5
**Hand RT**	DDR	233±29	224±25	.122	4
	CON	227±33	232±34		−2
**Knee ext**	DDR	28.9±8.1	29.5±7.8	.439	2
	CON	32.4±10.5	31.8±11.2		−2
**MET**	DDR	21.7±1.9	22.1±1.4	**.044**	2
	CON	21.4±2.3	21.0±1.5		−2
**Proprioception**	DDR	3.0±1.7	2.3±1.1	.489	23
	CON	2.2±0.9	2.4±1.5		−9
**TUG**	DDR	9.6±1.3	9.1±1.4	.843	5
	CON	9.8±1.4	9.3±1.8		5
**5 STS**	DDR	11.5±2.3	10.7±2.8	.430	7
	CON	10.8±2.4	10.3±2.1		5
**AST**	DDR	9.2±1.8	8.8±1.7	.423	4
	CON	9.3±4.7	9.0±2.1		3
**TMT B-A**	DDR	47±21	43±15	.443	9
	CON	61±36	74±61		−21
**TUG animals**	DDR	14.1±5.6	11.5±3.7	**.049**	18
	CON	11.9±2.9	12.0±3.5		−1
**INHIB**					
*time20trials*	DDR	51±17	42±7	.126	18
	CON	53±11	51±17		4
*time/trial*	DDR	2.5±0.8	2.1±0.3	.*094*	16
	CON	2.5±0.5	2.4±0.7		4
*errors*	DDR	1.0±1.9	0.9±1.3	.546	10
	CON	1.1±1.5	1.2±1.4		−9
**Icon-FES**	DDR	16.3±4.5	15.9±3.7	.648	2
	CON	17.6±5.6	17.2±5.0		2

a = positive scores indicate improvement; AP = antero-posterior; AST = alternate step test; CON = control group; CSRT = choice stepping reaction time; DDR = intervention group; Icon-FES = iconographical falls efficacy scale; Knee ext = knee extension strength; MET = Melbourne Edge Test; ML = medio-lateral; MT = movement time; PPA = Physiological Profile Assessment score; resp = response time; RT = reaction time; TMT B-A = difference between Trailmaking test B and A; TUG = timed up & go test; 5STS = five times sit-to-stand.

### Dose Response Effects

The level of game difficulty played at the end of the trial was significantly associated with improvement in PPA scores. Participants playing at the easy level at the completion of the trial showed no improvement in their scores. Participants playing at the hard level at the end of the intervention had significantly larger improvements in time to complete INHIB compared to those playing at the easy/medium levels (t = 2.444, df = 13, p = 0.030). No dose effects were observed for CSRT practice on the PPA, INHIB and CSRT outcome measures.

## Discussion

An eight week, home-based step pad training program produced improvements in several outcome measures associated with falls in higher functioning older people. The step training system proved feasible to administer and all participants included in the analysis could use the system, chose a variety of songs, changed game levels of difficulty and switched between CSRT and DDR tasks. All but one IG group participant enjoyed the step training and no adverse events related to the intervention were reported, suggesting step pad training is a safe mode of exercise for higher functioning older people.

### Effectiveness of the Intervention

Intervention participants improved both their step reaction times and movement times indicating improved central processing speed and movement velocity. The resultant overall improvement of 12% from the eight weeks of training is in agreement with a previous study of a voluntary step training regimen in older people that used a variety of step combinations and improved CSRT by a similar amount [12]. The mean improvement of 117 ms in the IG appears clinically meaningful as this is similar to the difference in CSRT times of 150 ms between multiple fallers and non-multiple fallers [23].

IG participants also recorded significantly improved PPA scores at the end of the intervention period. The average PPA score at baseline for this study sample was 1.75 indicating a moderate risk of falls [24]. The 34% reduction in PPA scores appears clinically meaningful, reducing the number of participants with a marked fall risk from 6 to 2 and increasing the number of participants having a mild or lower risk of falls from 3 to 6.

The reductions in PPA scores in the IG were primarily due to improved performance in postural sway and contrast sensitivity. Postural sway has been shown to be a predictor for falls in several studies [31], [32], [33], and it has been suggested that sway is a marker of balance control that is not reflexive but rather an experience-dependent skill to modulate the ankle musculature [34]. Improvements in sway may therefore be manifest in reduced fall risk in older people. It has also been reported that four weeks DDR play significantly reduces sway in young adults [35] further supporting our finding that a dynamic balance approach can lead to improvements of standing balance control.

We also found a significant between-group difference in contrast sensitivity due to a small improvement in the intervention and a small decline in the control group. The Melbourne Edge Test used for the contrast sensitivity assessment was administered under the same spatial and light conditions on both test occasions and has demonstrated high test-retest reliability (ICC = 0.81 (95%CI 0.70–0.88)) [24]. Previous research showing associations between computer game play and contrast vision provides support for this finding. Li et al. found that healthy young expert action video game players have superior contrast sensitivity compared to age- and gender-matched non-action game players using a different test paradigm [36], and Hale et al. recently reported that simple computer skills training improved contrast sensitivity in older people [37]. Similarly to action games in younger people intervention tasks in this study consisted of fast moving objects and required accurate, visually guided aiming actions.

In relation to cognitive measures, no improvements for the pen and paper-based trail making tests were found. However, we observed a between group difference in dual task ability as participants in the IG had faster TUG times while performing the verbal fluency task. Further, there was a trend indicating better performance of the IG in the INHIB stepping time. Both the measures combine motor and cognitive functions, namely divided and selective attention and executive function and may be more closely associated with daily activities and therefore more amenable to change. These specific cognitive functions have been consistently associated with falls [38] showing the potential benefits of a stepping intervention performed under a cognitive load. The nature of this game is to step at specific times rather than as fast as possible. In addition, the step sequence programmed into the system did not match the beat pattern inherent in each song. Participants were therefore required to pay close attention in order to play the game correctly and improve their scores. Further, participants had to inhibit their step response when ‘bombs’ drifted up on the screen in between the drifting arrows. This ‘go/no-go’ task under time pressure would have additionally increased the cognitive load of the game.

Some measures of physical function and fear of falling did not show significant differences between the intervention and control groups at the end of the eight-week intervention. For the physical parameters this may be due to ceiling effects as the participants were quite active and high-functioning, findings that are in agreement with previous studies undertaken in similar samples [12], [39]. Furthermore, this intervention may not have been specific enough to improve strength-related outcome measures. For the fear of falling measure this null finding may be due to the short-term intervention as previous exercise-based interventions that have been successful in reducing fear of falling have been of durations of at least 4 months [40], [41]. Furthermore, it might take longer for physical improvements to be reflected as a reduced fear of falling. No ceiling effect was observed for icon-FES scores (mean score  = 17 in a scale with a range 10 to 40).

### Feasibility of the Intervention in High-functioning Older People

The mode of delivery, a home-based, unsupervised program, was feasible, enjoyable and safe in high-functioning older people without mobility problems or cognitive impairment. The drop-out rate was only 13.5%, with 4 of 5 drop-outs either not starting the intervention or unable to finish the trial due to health reasons at the time of re-assessment. Participants who were not available for reassessment may have been frailer and lower functioning. However, the participant numbers were too small to explore this in more detail. Home-based exercise has been shown to be effective in reducing falls [42] and evidence suggests that training at home increases adherence of older adults to physical activity in the long-term [43]. This is an important public health issue as many benefits of exercise are lost soon after interventions cease [44]. Studies in young people have shown that compared to traditional modes of physical activity, exergames are perceived as more enjoyable [35], [45] and lead to higher adherence rates [46], [47]. Other findings suggest that playing action computer games decreases perceived pain through distraction [48] which may lead to higher intensities and better adherence when playing exergames compared to traditional modes of exercise [22]. Findings here indicate both, efficacy and acceptable adherence suggesting that a step pad exergame may offer a new exercise modality with the potential of preventing falls.

### Study Limitations

This study has a number of limitations. First, the sample size was possibly too small to detect changes in some of the secondary outcome measures including the tests of executive functioning and fear of falling. Second, one participant withdrew from the intervention because she was unable to use the system indicating that this approach might be unsuitable for people without sufficient skills in handling new technologies. Third, the sample comprised reasonably healthy and high functioning older people, so findings cannot be generalized to frail older people who may not be able to safely undertake the unsupervised program. However, it has recently been shown that a supervised group-based program combining DDR stepping with balance and strength training conducted in lower functioning people living in assisted living facilities achieved an adherence rate of 96% [49], indicating this approach may be feasible for many older people if adapted appropriately. Fourth, study participants were not blinded to group allocation. Although we asked members of the control group to maintain their usual activity levels, it is possible some may have increased their exercise levels during the trial period – a factor that may have biased results in favor for the control group. Finally, exercise participants regularly completed a CSRT task in addition to the DDR step training so the beneficial effects found need to be considered in light of this exercise ‘package’ and the effects of the two exercise components cannot be isolated.

### Conclusions

In summary, this pilot study found that short-term step pad training is safe and feasible to be administered at home and led to improvements in physical and cognitive parameters of fall risk in high functioning older people. Future studies with larger samples and intention-to-treat analysis need to confirm these findings and show if other outcomes such as fear of falling can be improved, whether this intervention undertaken over a longer period is able to prevent falls and if results can be transferred to other settings with lower functioning people.

## Supporting Information

Checklist S1 **CONSORT Checklist.**
(DOC)

Protocol S1 **Trial Protocol.**
(PDF)

## References

[1] Masud T, Morris RO (2001) Epidemiology of falls. Age Ageing 30: 3–7. 10.1093/ageing/30.suppl_4.3 11769786

[2] Anstey KJ, Wood J, Kerr G, Caldwell H, Lord SR (2009) Different cognitive profiles for single compared with recurrent fallers without dementia. Neuropsychology 23: 500–508. 19586213 10.1037/a0015389

[3] Lord SR, Ward JA, Williams P, Anstey KJ (1994) Physiological factors associated with falls in older community-dwelling women. J Am Geriatr Soc 42: 1110–1117. 7930338 10.1111/j.1532-5415.1994.tb06218.x

[4] Pijnappels M, Delbaere K, Sturnieks DL, Lord SR (2010) The association between choice stepping reaction time and falls in older adults–a path analysis model. Age Ageing 39: 99–104. 20015855 10.1093/ageing/afp200

[5] Tseng SC, Stanhope SJ, Morton SM (2009) Impaired reactive stepping adjustments in older adults. J Gerontol A Biol Sci Med Sci 64: 807–815. 19351694 10.1093/gerona/glp027 PMC2691798

[6] Lord SR, Fitzpatrick RC (2001) Choice stepping reaction time: a composite measure of falls risk in older people. J Gerontol A Biol Sci Med Sci 56: M627–632. 11584035 10.1093/gerona/56.10.m627

[7] Maki BE, McIlroy WE (2006) Control of rapid limb movements for balance recovery: age-related changes and implications for fall prevention. Age Ageing 35: 12–18. 10.1093/ageing/afl078 16926197

[8] Melzer I, Kurz I, Shahar D, Oddsson LI (2010) Do voluntary step reactions in dual task conditions have an added value over single task for fall prediction? A prospective study. Aging Clin Exp Res 22: 360–366. 21422793 10.1007/BF03324940

[9] Sherrington C, Whitney JC, Lord SR, Herbert RD, Cumming RG, et al (2008) Effective exercise for the prevention of falls: a systematic review and meta-analysis. J Am Geriatr Soc 56: 2234–2243. 19093923 10.1111/j.1532-5415.2008.02014.x

[10] Mansfield A, Peters AL, Liu BA, Maki BE (2010) Effect of a perturbation-based balance training program on compensatory stepping and grasping reactions in older adults: a randomized controlled trial. Phys Ther 90: 476–491. 20167644 10.2522/ptj.20090070

[11] Rogers MW, Johnson ME, Martinez KM, Mille ML, Hedman LD (2003) Step training improves the speed of voluntary step initiation in aging. J Gerontol A Biol Sci Med Sci 58: 46–51. 12560410 10.1093/gerona/58.1.m46

[12] Shigematsu R, Okura T, Nakagaichi M, Tanaka K, Sakai T, et al (2008) Square-stepping exercise and fall risk factors in older adults: a single-blind, randomized controlled trial. J Gerontol A Biol Sci Med Sci 63: 76–82. 18245764 10.1093/gerona/63.1.76

[13] Angevaren M, Aufdemkampe G, Verhaar HJ, Aleman A, Vanhees L (2008) Physical activity and enhanced fitness to improve cognitive function in older people without known cognitive impairment. Cochrane Database Syst Rev: CD005381. 10.1002/14651858.CD005381.pub2 18425918

[14] Liu-Ambrose T, Donaldson MG (2009) Exercise and cognition in older adults: is there a role for resistance training programmes? Br J Sports Med 43: 25–27. 19019904 10.1136/bjsm.2008.055616 PMC5298919

[15] Liu-Ambrose T, Donaldson MG, Ahamed Y, Graf P, Cook WL, et al (2008) Otago home-based strength and balance retraining improves executive functioning in older fallers: a randomized controlled trial. J Am Geriatr Soc 56: 1821–1830. 18795987 10.1111/j.1532-5415.2008.01931.x

[16] Nyman SR, Victor CR (2011) Older people's recruitment, sustained participation, and adherence to falls prevention interventions in institutional settings: a supplement to the Cochrane systematic review. Age Ageing 40: 430–436. 21502163 10.1093/ageing/afr016

[17] Nyman SR, Victor CR (2012) Older people's participation in and engagement with falls prevention interventions in community settings: an augment to the Cochrane systematic review. Age Ageing 41: 16–23. 21875865 10.1093/ageing/afr103

[18] Baranowski T, Buday R, Thompson DI, Baranowski J (2008) Playing for real: video games and stories for health-related behavior change. Am J Prev Med 34: 74–82. 18083454 10.1016/j.amepre.2007.09.027 PMC2189579

[19] Maddison R, Mhurchu CN, Jull A, Jiang Y, Prapavessis H, et al (2007) Energy expended playing video console games: an opportunity to increase children's physical activity? Pediatr Exerc Sci 19: 334–343. 18019591 10.1123/pes.19.3.334

[20] Graves LE, Ridgers ND, Williams K, Stratton G, Atkinson G, et al (2010) The physiological cost and enjoyment of Wii Fit in adolescents, young adults, and older adults. J Phys Act Health 7: 393–401. 20551497 10.1123/jpah.7.3.393

[21] Guderian B, Borreson LA, Sletten LE, Cable K, Stecker TP, et al (2010) The cardiovascular and metabolic responses to Wii Fit video game playing in middle-aged and older adults. J Sports Med Phys Fitness 50: 436–442. 21178930

[22] de Bruin ED, Schoene D, Pichierri G, Smith ST (2010) Use of virtual reality technique for the training of motor control in the elderly. Some theoretical considerations. Z Gerontol Geriatr 43: 229–234. 20814798 10.1007/s00391-010-0124-7

[23] Schoene D, Lord SR, Verhoef P, Smith ST (2011) A novel video game–based device for measuring stepping performance and fall risk in older people. Arch Phys Med Rehabil 92: 947–953. 21549352 10.1016/j.apmr.2011.01.012

[24] Lord SR, Menz HB, Tiedemann A (2003) A Physiological Profile Approach to Falls Risk Assessment and Prevention. Phys Ther 83: 237–252. 12620088

[25] Podsiadlo D, Richardson S (1991) The timed “Up & Go”: a test of basic functional mobility for frail elderly persons. J Am Geriatr Soc 39: 142–148. 1991946 10.1111/j.1532-5415.1991.tb01616.x

[26] Csuka M, McCarty DJ (1985) Simple method for measurement of lower extremity muscle strength. Am J Med 78: 77–81. 10.1016/0002-9343(85)90465-6 3966492

[27] Tiedemann A, Shimada H, Sherrington C, Murray S, Lord S (2008) The comparative ability of eight functional mobility tests for predicting falls in community-dwelling older people. Age Ageing 37: 430–435. 18487264 10.1093/ageing/afn100

[28] Lezak MD, Howieson DB, Lohring DW (2004) Neuropsychological assessment. New York: Oxford University Press.

[29] Schoene D, Smith ST, Delbaere K, Lord SR (2012) Poor performance in a test of selective attention, response inhibition and stepping is associated with falls in older people. J Aging Phys Activ 20: S186–187.

[30] Delbaere K, T. Smith S, Lord SR (2011) Development and Initial Validation of the Iconographical Falls Efficacy Scale. J Gerontol A Biol Sci Med Sci 66: 674–680. 21350244 10.1093/gerona/glr019

[31] Lajoie Y, Gallagher SP (2004) Predicting falls within the elderly community: comparison of postural sway, reaction time, the Berg balance scale and the Activities-specific Balance Confidence (ABC) scale for comparing fallers and non-fallers. Arch Gerontol Geriatr 38: 11–26. 14599700 10.1016/s0167-4943(03)00082-7

[32] Pfeifer M, Begerow B, Minne HW, Schlotthauer T, Pospeschill M, et al (2001) Vitamin D status, trunk muscle strength, body sway, falls, and fractures among 237 postmenopausal women with osteoporosis. Exp Clin Endocrinol Diabetes 109: 87–92. 11341304 10.1055/s-2001-14831

[33] Fernie GR, Gryfe CI, Holliday PJ, Llewellyn A (1982) The relationship of postural sway in standing to the incidence of falls in geriatric subjects. Age Ageing 11: 11–16. 7072557 10.1093/ageing/11.1.11

[34] Loram ID, Maganaris CN, Lakie M (2005) Human postural sway results from frequent, ballistic bias impulses by soleus and gastrocnemius. J Physiol 564: 295–311. 15661824 10.1113/jphysiol.2004.076307 PMC1456055

[35] Brumels KA, Blasius T, Cortright T, Oumedian D, Solberg B (2008) Comparison of Efficacy Between Traditional and Video Game Based Balance Programs. Clin Kinesiol 62: 26–31.

[36] Li R, Polat U, Makous W, Bavelier D (2009) Enhancing the contrast sensitivity function through action video game training. Nat Neurosci 12: 549–551. 19330003 10.1038/nn.2296 PMC2921999

[37] Hale LA, Waters D, Herbison P (2012) A randomized controlled trial to investigate the effects of water-based exercise to improve falls risk and physical function in older adults with lower-extremity osteoarthritis. Arch Phys Med Rehabil 93: 27–34. 21982325 10.1016/j.apmr.2011.08.004

[38] Hsu CL, Nagamatsu LS, Davis JC, Liu-Ambrose T (2012) Examining the relationship between specific cognitive processes and falls risk in older adults: a systematic review. Osteoporos Int online first. 10.1007/s00198-012-1992-z PMC4476839 22638707

[39] Taylor D, Hale L, Schluter P, Waters DL, Binns EE, et al (2012) Effectiveness of Tai Chi as a Community-Based Falls Prevention Intervention: A Randomized Controlled Trial. J Am Geriatr Soc 60: 841–848. 22587850 10.1111/j.1532-5415.2012.03928.x

[40] Cyarto EV, Brown WJ, Marshall AL, Trost SG (2008) Comparative effects of home- and group-based exercise on balance confidence and balance ability in older adults: cluster randomized trial. Gerontology 54: 272–280. 18787321 10.1159/000155653

[41] Wolf SL, Barnhart HX, Kutner NG, McNeely E, Coogler C, et al (1996) Reducing frailty and falls in older persons: an investigation of Tai Chi and computerized balance training. Atlanta FICSIT Group. Frailty and Injuries: Cooperative Studies of Intervention Techniques. J Am Geriatr Soc 44: 489–497. 8617895 10.1111/j.1532-5415.1996.tb01432.x

[42] Gillespie LD, Robertson MC, Gillespie WJ, Lamb SE, Gates S, et al.. (2009) Interventions for preventing falls in older people living in the community. Cochrane Database Syst Rev: CD007146. 10.1002/14651858.CD007146.pub2 19370674

[43] Ashworth NL, Chad KE, Harrison EL, Reeder BA, Marshall SC (2005) Home versus center based physical activity programs in older adults. Cochrane Database Syst Rev: CD004017. 10.1002/14651858.CD004017.pub2 PMC6464851 15674925

[44] Wagner EH, LaCroix AZ, Grothaus L, Leveille SG, Hecht JA, et al (1994) Preventing disability and falls in older adults: a population-based randomized trial. Am J Public Health 84: 1800–1806. 7977921 10.2105/ajph.84.11.1800 PMC1615188

[45] Plante TG, Aldridge A, Bogden R, Hanelin C (2003) Might virtual reality promote the mood benefits of exercise? Computers in Human Behavior 19: 495–509.

[46] Annesi JJ, Mazas J (1997) Effects of virtual reality-enhanced exercise equipment on adherence and exercise-induced feeling states. Percept Mot Skills 85: 835–844. 9399288 10.2466/pms.1997.85.3.835

[47] Rhodes RE, Warburton DER, Bredin SSD (2009) Predicting the effect of interactive video bikes on exercise adherence: An efficacy trial. Psychol Health Med 14: 631–640. 20183536 10.1080/13548500903281088

[48] Raudenbush B, Koon J, Cessna T, McCombs K (2009) Effects of playing video games on pain response during a cold pressor task. Percept Mot Skills 108: 439–448. 19544949 10.2466/PMS.108.2.439-448

[49] de Bruin ED, Reith A, Dörflinger M, Murer K (2011) Feasibility of Strength-Balance Training Extended with Computer Game Dancing in Older People; Does it Affect Dual Task Costs of Walking? J Nov Physiother 1: 104.

