# Supplementary material for: A Randomized Controlled Pilot Study of Home-Based Step Training in Older People Using Videogame Technology
Source: PLoS One. 2013 Mar 5;8(3):e57734. doi: 10.1371/journal.pone.0057734 (PMC3589451; doi:10.1371/journal.pone.0057734)
Supplement: Protocol S1 — Trial Protocol. (PDF) [file pone.0057734.s002.pdf]

SECTION 9: DESCRIPTION OF PROJECT
(refer to the National Statement on Ethical Conduct in Research Involving Humans, p. 13)

This section is obligatory

9.1 Describe the project using lay terms wherever possible, including the aims, hypotheses, research plan and potential significance. Where relevant, provide the projected number, sex, and age range of participants (including inclusion/exclusion criteria). You must satisfy the HREC that the study is scientifically valid and conducted in accordance with the accepted ethical principles governing research involving humans.
The description must be no longer than 2 pages and must be in a font size of at least 10 points.

Aims:
- To determine the effects of a step training intervention on protective and volitional stepping and key indicators of fall risk.
- To determine the effects of the program on secondary measures including muscle strength and power and leaning balance.
- To establish predictors of adherence to the program.

Design: This study will involve installation of our step training system into the homes of older adults for an investigation of the potential benefits of a home-based program of step training. Older adults will be randomised into either a step training or control group. We will assess whether a 24-week period of training using the system has a significant impact on stepping ability and fall risk.

Participants: will randomly draw 100 participants (50 women) from the prospective study described above (NHRMC Project Grant 510110). The eligibility criteria will be the same as for Study 1.

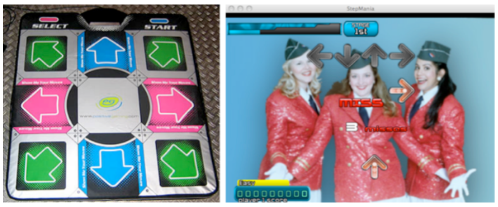

Figure 1. Standard dance pad input device (left) for interacting with dance-style video games such as StepMania (right).

Step training system: Interactive, user input devices such as dance pads (Fig 1) are ideally suited for development of a low-cost, interactive method for training stepping ability: "Dance pad" games, where repetitive medio-lateral and anterior-posterior steps are required offer a novel, yet effective, technique for training stepping ability in older adults. These games are played on a flat dance pad sensor (Fig 2, left) that measures about 1 square metre and has between four and eight step panels (arrows). The pad is connected to a visual display screen such as a television that provides step direction instructions to the player via a system of scrolling arrows that typically rise slowly from the bottom to the top of the screen. As the arrows scroll up to the top of the screen, they cross over a set of four corresponding arrow silhouettes. The player must step on the corresponding mat arrow as the scrolling arrow crosses its silhouette. Sequences of steps can range in difficulty from simple marching or walking patterns to those with varied rates and irregular patterns that challenge co-ordination and attention. We have developed a dance mat video game that is appropriate for the functional level and interests of older adults.

Outcome measures: All assessments will be conduced immediately before and at the end of the 24 week trial will be conducted at a laboratory at POWMRI. Choice stepping reaction time, waist pull perturbation responses and PPA fall risk index scores will comprise the primary outcome measures.

Choice stepping reaction time - a composite assessment of balance and reaction time, which has previously been found to be a good predictor of falls in older adults [1]. This measure will provide information regarding individual's volitional stepping performance. Total CSRT (composed of initiation and transfer reaction times) will comprise the primary CSRT outcome measure.

Waist pull perturbation - Participants will be pulled unexpectedly in different directions, with a randomly presented series of 3 forces, 3 displacements and 3 velocities. Participants stand with four very light but stiff cords attached to a broad belt that is fixed securely about the pelvis (Fig 2). The cords have an in-series force transducer (z), connected to a tensiometer mounted on a linear motor (M), so that the subject can be pulled forwards, backwards and laterally. Subjects are told to stand "at ease" and not voluntarily intervene. Movements (displacements, velocities, accelerations) in response to force-controlled perturbations, in addition to the forces underfoot and muscle activity responsible for these observed movements, are quantified. Other outcomes that can be measured using this technique include COM position at step initiation, COM peak velocity, step time, step position, number of steps taken and whether any foot collisions occur in response to lateral perturbations. The primary outcome measure from the waist-pull perturbation test will be the force threshold for stepping.

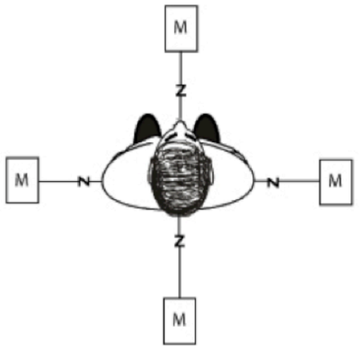

Figure 2. Four linear motor setup for AP and ML waist pull perturbations

Falls Risk - Physiological profile assessment (PPA) - Falls Risk will be determined using the short-form PPA which contains five validated measures of physiological function [2]. In multivariate models, weighted contributions from these five variables provide a falls risk score (primary outcome) that can predict community-dwelling people at risk of multiple falling with 75% accuracy in a 12-month period. The five short-form PPA tests are:

1. Visual Contrast Sensitivity - assessed using the Melbourne Edge Test.
2. Proprioception - measured using a lower limb-matching task. Errors in degrees are recorded using a protractor inscribed on a vertical clear acrylic sheet placed between the legs.
3. Quadriceps strength - measured isometrically in the dominant leg, while participants are seated with the hip and knee flexed to 90 degrees.
4. Simple reaction time - measured using a light stimulus and a finger-press as the response.
5. Postural sway (maximal anterior-posterior and lateral displacement) - measured using a sway meter recording displacements of the body at the level of the pelvis. Testing is performed standing on a foam rubber mat with eyes open.

Randomisation: Participants will be randomised into either a step training or control group using a computer generated random number schedule with randomly permuted block sizes of 6–10. Randomisation will be done centrally by Dr Sherrington who will not be directly involved in participant recruitment (i.e. a concealed randomisation system). There will be 50 participants per group.

Intervention: Following recruitment and randomisation, a home visit to each participant in the training group will be conducted to establish an appropriate location within the home for training and address any possible physical or technical impediments to system use. A rigid frame to surround the dance pad will be supplied as a safety measure. During this first home visit participants in the training group will receive a lesson on system use and will be asked to suggest any song titles or visual images (e.g. pictures of grandchildren) they would like incorporated into their personalised step training system. By encouraging an active role by older adults in development of the system, we aim to further promote adherence to exercise training programs.

Home visits by an exercise trainer will be conducted in weeks 1, 2, 3,6 and 12 to encourage ongoing system use, monitor system operation and to discuss any issues which have arisen during previous step training sessions, advise on progression of the step training routines and encourage adherence. Follow-up phone calls to participants will be made monthly during the 24 week study period to encourage ongoing participation in the program rates.

During the intervention, participants will self-select the rate of progression of difficulty and may repeat lessons / songs to improve their “grade” or try new ones. Participants in the training group will be encouraged to engage in at least three 30 minute sessions per week for each of the twenty four weeks of the intervention with a recommended goal of at least 4 sessions and 120 minutes per week of system use. The intensity and type of the step training exercises can be adjusted as performance improves to ensure that the intervention remains challenging.

Adherence, Progression and Adverse Events: Using the interactive step training system deployed into the homes of participants in the step training group, we will also be able to monitor the progression of reaction time indices recorded by the system software. The step training system will also be able to record date and time of use as well as scores of the participants. Adherence rates will be monitored by home exercise diaries kept by participants and compared against usage data automatically recorded by the step training system. Participants will also be asked to identify reasons for adoption and adherence as well as non-adoption and non-adherence. These data will be analysed in a similar manner to the one used in a previous study by Prof Lord [3]. These data will be used to measure adherence to the step training intervention. We will monitor any adverse events due to system use such as falls or sore muscles.

Control group: Participants in the control group will receive usual care so will not be disadvantaged by being in the study. In addition, they will receive an education booklet about falls prevention.

References:

[1] Lord SL and Fitzpatrick RC. 2001. Choice stepping reaction time: A composite measure of falls risk in older people. Journal of Gerontology: Medical Sciences. 56A(10), M627-632

[2] Lord SR, Menz HB et al. 2003. A physiological profile approach to falls risk assessment and prevention. Phys Ther, 83(3), 237-252

[3] Williams, P, Lord SR: Predictors of adherence to a structured exercise program for older women. Psych & Aging. 1995; 10(4): 617-24
